# Vertical Transmission of Sindbis Virus in *Culex* Mosquitoes

**DOI:** 10.3390/v14091915

**Published:** 2022-08-30

**Authors:** Emma Dahl, Linnea Öborn, Viktoria Sjöberg, Åke Lundkvist, Jenny C. Hesson

**Affiliations:** Department of Medical Biochemistry and Microbiology/Zoonosis Science Center, Uppsala University, Box 582, 751 23 Uppsala, Sweden

**Keywords:** alphavirus, Sweden, overwintering, temperate region, vector, arbovirus, viral persistence, winter season

## Abstract

**Simple Summary:**

The transmission of vector-borne viruses requires arthropod vectors that are actively seeking for new blood-meal hosts. In regions where vectors do not blood-feed for prolonged periods, the persistence of a virus depends on alternative mechanisms of transmission, such as the transmission of a virus from an infected arthropod mother to her offspring, via so-called vertical transmission. Far less is known about this type of transmission than about transmission between vertebrate hosts, and it is often viewed as rarely occurring. Sindbis virus is one of many mosquito-borne viruses that originates in the tropics and has become introduced and established in temperate regions. In its northern range, the virus must persist through several months of winter when its mosquito vectors are inactive. In this study, we investigated the vertical transmission of Sindbis virus, both experimentally and in the field, and found evidence from the field that it does occur but with conflicting results in the experiments. This new knowledge highlights factors which are necessary for tropical viruses to establish in temperate regions.

**Abstract:**

Vertical transmission (VT) is a phenomenon of vector-borne diseases where a pathogen is transferred from an infected arthropod mother to her offspring. For mosquito-borne flavi- and alphaviruses, VT is commonly viewed as rare; however, both field and experimental studies report on vertical transmission efficiency to a notably varying degree. It is likely that this reflects the different experimental methods used to test vertical transmission efficiency as well as differences between virus–vector combinations. There are very few investigations of the VT of an alphavirus in a *Culex* vector. Sindbis virus (SINV) is an arthritogenic alphavirus that utilizes *Culex* species as main vectors both in the summer transmission season and for its persistence over the winter period in northern latitudes. In this study, we investigated the vertical transmission of the SINV in *Culex* vectors, both in the field and in experimental settings. The detection of SINV RNA in field-collected egg rafts and emerging adults shows that vertical transmission takes place in the field. Experimentally infected females gave rise to adult offspring containing SINV RNA at emergence; however, three to four weeks after emergence none of the offspring contained SINV RNA. This study shows that vertical transmission may be connected to SINV’s ability to persist throughout northern winters and also highlights many aspects of viral replication that need further study.

## 1. Introduction

Arthropod-borne viruses, arboviruses, cause disease across the globe and throughout all climate zones. The transmission of arborviruses is dependent on an active arthropod vector that can transfer the virus to a vertebrate host, via so-called horizontal transmission. In some regions, vector populations will go through periods of low activity where blood feeding ceases due to environmental circumstances, and horizontal transmission is decreased to a minimum or is completely halted. In tropical regions, such environmental changes can be, e.g., the dry season, which offers less habitat for the immature stages of some vectors. Temperate regions are marked by a winter season with temperatures allowing limited or completely halted vector activity. In the most northern regions, vectors need to go into hibernation for several months before they can emerge in the spring and rebuild the population and potentially also restart a new arboviral transmission season. Mosquito-borne arboviruses affected by vector hibernation include both flaviviruses, e.g., West Nile virus (WNV) and Japanese encephalitis virus (JEV), and alphaviruses, e.g., Sindbis virus (SINV).

These three viruses, WNV, JEV, and SINV, all have a tropical origin but have also become established in temperate regions. They are all transmitted by mosquitoes of the genus *Culex*, and it has been shown that they can persist throughout northern winters in hibernating adult *Culex* females [1,2,3]. These females emerge as adults in late summer, mate, and feed on nectar before they enter a suitable hibernation site which will provide shelter with temperatures close to freezing, such as, e.g., cellars or caves [4,5]. Many studies on hibernating *Culex* female populations in North America, Europe, and Asia have shown that the vast majority of these females have never blood-fed when they enter hibernation. Instead, their first blood meal is taken after they exit hibernation in the spring [1,3,5,6]. In a previous study on SINV in hibernating *Cx. pipiens* females in Sweden, we showed that several virus-infected individuals had not previously taken a blood meal [3]. A very important question followed: how did these females become infected?

The most common hypotheses are that these females became infected either through the process of vertical transmission, i.e., when an infected female transmits the virus to her offspring, or through venereal transmission, i.e., during mating. Vertical transmission is a prerequisite for venereal transmission as males do not blood-feed and is the most commonly investigated type of alternative virus transmission. Vertical transmission has been shown experimentally in the laboratory for both alphaviruses and flaviviruses, transmitted by both *Culex* and *Aedes* vector species. However, the experimental designs, as well as the results, are inconsistent, and in general, vertical transmission is considered to occur at low efficiency for these viruses [7].

Despite this, the fact that these arboviruses do persist endemically in regions where long winters interrupt vector activity and that viruses have been detected in non-blood-fed hibernating vectors, strongly suggests that alternative methods of transmission must be present and also efficient enough to establish a new season of virus transmission every year. In northern Europe, SINV is endemic and can be detected in the vector population (*Cx. pipiens*, *Cx. torrentium, Aedes cinereus, Ae. rossicus,* and *Culiseta morsitans*) every year [8,9]. SINV is a cause of disease in humans in late summer and early autumn, where infections may present with fever, rashes, and arthralgia, which may become chronic [10]. A phylogeographic study of SINV in northern Europe has shown that the virus was introduced from central Africa sometime in the 1920s and that it has persisted in the region since [11]. The main vertebrate hosts of SINV are passerine birds, and it has been hypothesised that the birds were the means of transport for SINV from Africa to Europe [12,13]. Thus, SINV is one example of a tropical virus that has successfully been introduced to a temperate climate zone and adapted to northern vector- and host species, as well as climate conditions. As we previously detected SINV in hibernating, non-blood-fed *Cx. pipiens* females [3], this study investigates the vertical transmission of SINV in *Culex* mosquitoes, both in a controlled laboratory experiment and in the field.

## 2. Methods

### 2.1. Field Studies

To estimate SINV vertical infection rates in the field, collections of *Cx. pipiens/torrentium* egg rafts were made within a 10 m^2^ area in Ovanåker commune, Sweden (61.34366, 16.06638), where we previously reported on high SINV occurrence in overwintering *Cx. pipiens* [3]. Hay infusion was prepared by mixing water, hay, beer yeast (0.05%), and lactalbumin (0.05%) and left to ferment for approximately four weeks prior to the sampling of the egg rafts, using 12 five-litre plastic buckets. Egg rafts were sampled on the 9–11 August 2020, and on the 15–16 August 2021. In 2020, the egg rafts were put in separate water containers and allowed to develop into adults. One larva per egg raft was identified to the species using a molecular assay separating *Cx. pipiens pipiens* and *Cx. torrentium* [14]. Pupae of the same species were pooled together and allowed to hatch in a netted cage where they were kept at approximately 21 °C and 60% humidity and a photoperiod of 18 h of light and 6 h of darkness, for four to six weeks before being killed and analysed for SINV; see below. During incubation, the mosquitoes were fed 10% sugar solution. In 2021, the egg rafts were collected and immediately frozen at −20 °C and, after a few days, stored at −80 °C until analysis.

### 2.2. Experimental Methods

Experiments were performed on an autogenous (i.e., capable of laying eggs without a blood meal) colony of *Cx. pipiens molestus*, a biotype of SINV vector species *Cx. pipiens* that feeds more efficiently in laboratory conditions. The females (F0) were starved for 28 h before being offered SINV (strain 09-M-358-5 or 09-M-991-1) at a titre of approximately 5*10^6PFU/mL diluted in defibrinated horse blood supplemented with 5% sugar or 10% sugar solution with 5% blue food dye added and added to a cotton pad. The mosquitoes were allowed to feed for two hours in the dark before the fed mosquitoes were sorted out based on their colouring and incubated for 17–20 days at 21 °C and a photoperiod of 18 h of light and 6 h of darkness. The incubation temperature and time were chosen based on natural conditions occurring during Swedish summers and prior pilot studies that have shown that this colony of *Cx. pipiens molestus* both survives and develops a disseminated infection (and thus has the potential to transmit the virus to their eggs) after ingesting SINV through both blood and sugar feeding under these conditions (unpublished). After incubation, some of the surviving females were placed in individual cups with hay infusion to encourage egg laying. The rest of the females were pooled and placed in bigger infection pots, also with hay infusion to encourage egg laying. The cups and pots were inspected daily for egg rafts.

All laid egg rafts from individually kept females were placed in separate containers and F0-females were removed within two days after egg laying and frozen for analysis of infection status. Only the egg rafts from females confirmed to be SINV-infected were continued in the experiment, while the egg rafts and any hatched offspring from uninfected females were disposed of. For the females kept in pools, all the egg rafts were kept and reared as these could not be assigned to an individual female. Pooled F0 females were removed after 12 days and analysed for infection status.

Containers with hatched egg rafts were checked daily for pupae, which were placed in batch-specific netted cups where they were allowed to emerge as adults. Some of the emerging F1 adults were removed within two days after emergence and frozen for later analysis, while the rest were kept for 20–27 days at 21 °C before being frozen for later analysis.

### 2.3. SINV Detection and Specificity of the Assay

All samples were stored at −80 °C until analysis. The mosquitoes and field-collected egg rafts were homogenised in 300 μL of PBS supplemented with 20% FBS, 1% PenStrep, and 1% Amphotericin B. Homogenisation was performed using either a pestle or by bead-beating with two stainless-steal beads and a QIAGEN TissueLyser II (Qiagen, Hilden, Germany). Viral RNA was extracted from the homogenate using the QIAamp Viral RNA mini kit according to the manufacturer’s instructions (Qiagen, Hilden, Germany).

A SINV RTqPCR was used to detect viral RNA, using primers targeting the non-structural protein 1, Fwd: GGTTCCTACCACAGCGACGAT, Rev: TGATACTGGTGCTCGGAAAACA and probe: FAM-TTGGACATAGGCAGCGCA-MGBNFQ, and 5 μL of RNA with the Quanta qScript One-Step System (Quanta Biosciences, Beverly, MA, USA), following the manufacturer’s instructions [15]. All reactions were run in duplicates and negative and positive controls were included in all runs. Extracted and diluted RNA from a SINV stock (strain 09-M-991-1) was used as a positive control. To evaluate the specificity of the method and establish a cut-off Ct value, RNA from 46 uninfected *Cx. pipiens molestus* colony females was extracted and used with the RTqPCR, evaluating the use of both the 5 and the 10 μL sample RNA input.

## 3. Results

### 3.1. Specificity of the Assay

When the RTqPCR was used with the 10 μL input, 13 out of 46 SINV-negative mosquitoes had a Ct value of 37 or more, albeit five of these barely crossed the baseline, which was set at 230 relative fluorescence units (RFU). When the sample input was decreased to 5 μL of RNA, 8 out of the 46 negative control mosquitoes had a Ct value of 38,74 or higher, using the same positive control and baseline threshold (Figure S1). Accordingly, a sample above these limits cannot be considered reliably positive as an unspecific amplification of SINV-negative mosquitoes may occur. Based on this, we set a cut-off value at Ct < 38 for our analysed samples to be regarded as positive, either in both duplicate wells or in a confirmatory additional run. Notably, the RTqPCR did not consistently amplify the same negative control samples; thus, unspecific amplification was random and not caused by any specific material present in individual mosquitoes.

### 3.2. Vertical Transmission in the Field

Vertical transmission of SINV in the field-sampled *Culex* could be detected during both of the two consecutive years investigated. In 2020, five *Culex* egg rafts were collected, from which three were from *Cx. torrentium* and two were from *Cx. pipiens pipiens*. A total of 94 adult *Cx. torrentium* (39 females, 55 males) emerged and were analysed by RTqPCR after being kept four to six weeks at 21 °C. Six of these females were found positive for SINV RNA (Ct values between 28 and 32). Of the 21 adult *Cx. pipiens pipiens* (10 females, 11 males) that emerged and were analysed for SINV by RTqPCR after being kept four to six weeks at 21 °C, none were positive. In 2021, 20 *Culex* spp egg rafts were collected and two contained SINV RNA (Ct values 35 and 36).

### 3.3. Experimental Vertical Transmission

From the 185 females that fed on SINV-spiked sugar solution, 149 survived the incubation and were given the opportunity to oviposit, either in individual cups (*n* = 73) or in three pools of 25, 25, and 26 individuals, respectively. From the individually kept F0 females, 25 laid eggs, and from the pooled mosquitoes, more than 20 egg rafts were laid. Twelve of the individually kept F0 females that laid egg rafts were confirmed to have been infected by SINV. From these infected F0 females, seven (58%) egg rafts hatched, while five (42%) egg rafts did not hatch (Table 1). From the 76 pooled F0 females, approximately 14 egg rafts were laid, and all the pools contained F0 females with confirmed SINV infection (Table 2).

From the 54 females that fed on SINV-spiked blood, 51 survived the incubation and were given the opportunity to oviposit, either in individual cups (*n* = 35) or in a pool of 16 individuals.

From the individually kept F0 females, 18 laid eggs. From the pooled mosquitoes, more than six egg rafts were laid. Seven of the individually kept F0 females that laid egg rafts were confirmed to have been infected by SINV. From these infected females, four (57%) egg rafts hatched while three (43%) egg rafts did not hatch (Table 1). From the 16 pooled F0 females, approximately six egg rafts were laid, and all the pools contained F0 females with confirmed SINV infection (Table 2).

The F1 offspring from infected females that were kept individually were sampled either within two days after emergence or after incubation for 20–27 days at 21 °C. From the 67 F1 adults originating from the 12 F0 females infected with SINV-spiked sugar solution and sampled within two days after emergence, 16 (23%) contained SINV RNA (Table 1). In this group, all 12 clutches contained at least one individual that tested positive for SINV RNA (Ct values 32–37), with an average filial infection rate of 31%. Interestingly, none of the 23 F1 adults originating from the F0 females infected with SINV-spiked blood and sampled after 20–27 days of incubation contained SINV RNA. Similarly, none of the 116 adult F1 offspring originating from the pooled F0 females infected by either sugar or blood spiked with SINV and also sampled after 20–27 days of incubation contained SINV RNA, despite the presence of F0 females with confirmed infection in all pools (Table 2).

In addition, non-hatched egg rafts could be rescued to a limited extent from both individually kept females and pools. Two out of four analysed egg rafts from individually kept females that had fed on SINV-infected blood tested positive for SINV RNA (Ct values 34 and 37). From the pooled females that had fed on SINV-infected blood, one out of two egg rafts tested positive for SINV RNA (Ct value 36).

## 4. Discussion

Vertical transmission is often considered a prerequisite for arboviruses’ ability to persist through adverse periods inside their mosquito vectors. As we previously detected SINV in non-blood-fed hibernating *Culex* females [3], the current study investigates the vertical transmission of SINV in *Culex* females, both in the field and in laboratory experiments. In our field material, sampled in the same region as the SINV-infected hibernating females, only 5 (year 2020) and 20 (year 2021) egg rafts were collected, and despite the different analysing strategies used for the two years, SINV was detected in six adult offspring and two egg rafts, respectively. As the emerging adults from the three collected egg rafts were pooled, we cannot know if the six individuals originated from either one, two, or three *Cx. torrentium* egg rafts. Regardless, supported by previous findings [3], this indicates a high SINV prevalence in *Culex* mosquitoes in this region and also that vertical transmission is occurring.

The laboratory experiments on vertical transmission confirmed that SINV RNA could be detected in the egg rafts originating from the infected F0 females and that some of their F1 progeny contained SINV RNA when they were sampled as newly emerged adults. In our experiments, the egg development of the F0 females occurred before they had the chance to develop a disseminated infection. This experimental set-up was chosen due to inefficient blood feeding, a common trait for *Culex* mosquitoes in laboratory settings, and the difficulty in obtaining repeated blood feeding and repeated gonotrophic cycles. Thus, the infection of the eggs likely took place in the oviduct at oviposition, the so-called transovum transmission. Such transmission is independent of gonotrophic cycle, unlike the transovarial transmission that occurs though infection of the female germinal tissues, with increased vertical transmission potential with the female age/gonotrophic cycle as a consequence. Whether one or both types of vertical transmission of SINV occur in the field remains to be studied.

Surprisingly, in the group where the F1 adults were kept for another 20–27 days, none of the offspring contained detectable SINV RNA. Similar results have been reported for Zika virus, where virus prevalence was significantly higher in three-day-old offspring of infected mothers than in the seven- and fourteen-day-old offspring [16]. A potential explanation for this could be that the virus to some extent infects the eggs and the developing mosquito but that it is not enough for efficient replication in the emerging adult. The detected RNA could thus be from fragmented virus particles that have been broken down after a few weeks of incubation. However, our results indicate that replication of SINV can take place after emergence as the Ct values for the F1 adults (Ct 28–32) originating from the field-collected egg rafts and kept for four to six weeks were lower than for the field-collected eggs (Ct 35–36), as well as for the newly emerged F1 adults originating from the experimentally infected mothers (Ct 32–37).

Another potential explanation is that the two experimental groups of individually kept females in the present study differed by means of infection, where the first group (sampled at emergence) originated from females that had fed on SINV-spiked sugar, and the second group (sampled after 20–27 days) originated from females that had fed on SINV-spiked blood. Blood feeding is the natural route of infection, while infection through sugar feeding is used as a way to infect mosquitoes that are not efficiently blood-feeding in a laboratory setting, which is often the case with *Culex* spp. We found that both routes of infection give comparable infection rates; however, infection load, measured as the Ct value, appears to be higher after feeding on SINV-spiked blood than on SINV-spiked sugar (unpublished). Whether this can be a reason for our different results for the F1 adults sampled immediately after emergence and the F1 adults sampled after incubation needs to be further investigated.

Nevertheless, the detection of SINV RNA in adult offspring originating from field-collected egg rafts, four to six weeks after emergence, indicates that SINV can replicate and persist in the F1 generation. Studies on both alpha- and flaviviruses indicate that potential triggers, such as temperature and metabolic changes inside the mosquitoes, may be needed for the activation of viral replication [17,18]. The influence of such triggers on viral replication is, however, not well studied and also not controlled for in our experiments.

Field studies of Chikungunya virus (CHIV), the most studied alphavirus, as well as several other alphaviruses, such as SINV, Ross River virus, and Western equine encephalitis virus, imply that vertical transmission does occur in nature [19,20,21,22,23]. Experimental studies of CHIV report very different vertical transmission rates: from absent or low [24,25,26] to efficient and even persisting for five successive generations [27]. Thus, although the experimental results vary greatly, the field data indicate that vertical transmission of alphaviruses does take place in nature [28]. An important difference between CHIV and SINV is that the former utilizes a vector species from another mosquito genus (*Aedes*). Studies on *Culex* mosquitoes are largely limited to flaviviruses and also report that vertical transmission can be detected in the field, but with substantial variation in the vertical transmission rates estimated in the experimental settings [29,30,31,32].

The big differences in vertical transmission rates reported in the literature may be attributed to different experimental designs (pooled versus individually kept F0 females), the different viruses and viral families investigated (alphaviruses and flaviviruses), and the mosquito species infected (*Aedes* and *Culex* species). However, it is also likely that the viral detection technique used has a large impact on the estimates of the infection rates. Most older studies use live animals or cell systems to analyse infectivity, while newer studies often use the detection of viral RNA through quantitative PCR. Cell-based methods, such as plaque tests that detect infectivity and/or titrate infectious virus in an artificial system, have the advantage of detecting functional virions but the disadvantage of lower sensitivity as they are not the natural target for the virus. Molecular methods are much more sensitive but only provide measurements of the RNA copy number, which may originate from non-infectious, defect virus particles, as well as traces of old viral RNA that have persisted over time [18]. Depending on the virus, the ratio between the viral titre and the RNA copy number may be very low [16,33,34]. Thus, PCR detection is not necessarily indicative of the presence of infectious virus. It is therefore important to highlight that in the current study, we only detected SINV RNA by PCR and did not attempt detection of infectivity. These results raise many additional questions on viral remnants versus active viral particles and highlight the need for more studies on how viral replication in the mosquito is affected by different stimuli, under ecologically relevant conditions.

The results presented here support vertical transmission as a possible mechanism for infecting hibernating *Culex* females with SINV, enabling a route of viral persistence over winter [3]. Infected females that exit hibernation would take their first blood meals from bird hosts during spring and re-initiate transmission between vectors and hosts, leading to a subsequent increase in SINV prevalence during the summer season [9,13]. Viral traits that enable persistence during the winter period are probably a necessity for an arbovirus to become established and endemically occurring in a temperate region, as phylogeographic studies suggest that it is unlikely that the reintroduction of arboviruses, such as SINV and WNV, from tropical regions to temperate regions occurs every year [11,35,36]. The ability to transmit vertically could be one such trait and could possibly evolve as an adaptation when the virus encounters new vector species and environmental conditions, similar to other recognised viral adaptations to vectors in new regions [37,38,39]. Another trait could be to transmit venereally, from vertically infected males to uninfected females. Studies of venereal transmission are limited, but experimental studies of SINV and CHIV in *Aedes* species have both shown approximately 10% transmission efficiency [40,41]. Potentially, these two alternative routes of infection, although not very efficient, could together account for the number of infected hibernating females necessary to start a new transmission cycle of virus in the spring.

## Figures and Tables

**Table 1:** Individually kept *Culex pipiens molestus* females that were confirmed to be infected by SINV and laid eggs.

F0 Female ID ^1^	Egg Rafts Hatched	Number of F1 Adults	Age of F1	Number of SINV-Positive F1 Adults	Sex of Infected F1 Adults ^2^
**B2**	yes	3	20–27 days	0	NA
**B6**	no	NA	20–27 days	NA	NA
**B18**	yes	10	20–27 days	0	NA
**B20**	no	NA	20–27 days	NA	NA
**B22**	yes	3	20–27 days	0	NA
**B24**	no	NA	20–27 days	NA	NA
**B31**	yes	7	20–27 days	0	0
**1**	yes	7	0–2 days	1	F
**2**	no	NA	0–2 days	NA	NA
**3**	yes	5	0–2 days	3	2F, 1M
**4**	yes	3	0–2 days	1	F
**8**	no	NA	0–2 days	NA	NA
**12**	no	NA	0–2 days	NA	NA
**16**	no	NA	0–2 days	NA	NA
**17**	yes	30	0–2 days	3	2F, 1M
**18**	no	NA	0–2 days	NA	NA
**20**	yes	10	0–2 days	5	4F, 1M
**22**	yes	6	0–2 days	1	M
**23**	yes	6	0–2 days	2	1F,1M

NA: not applicable; ^1^ ID with a number followed by the letter B indicates that the female was infected through blood feeding; ID with only a number indicates that the female was infected through sugar feeding; ^2^ F: female; M: male.

**Table 2:** Pooled *Culex pipiens molestus* females and their offspring produced.

Pool ID ^1^	Number of F0 Females	Number of Females with ConfirmedSINV Infection	Number of Egg Rafts ^2^	Number of F1 Adults
**1B**	16	16	≈6	9
**2**	25	16	≈4	25
**3**	25	23	≈5	30
**4**	26	25	≈5	52

^1^ ID with a number followed by the letter B indicates that the female was infected through blood feeding; ID with only a number indicates that the female was infected through sugar feeding. ^2^ The number of egg rafts laid by pooled females was estimated as individual egg rafts were difficult to observe.

## Supplementary Materials

The following supporting information can be downloaded at: https://www.mdpi.com/article/10.3390/v14091915/s1, Figure S1: amplification of uninfected negative control mosquitoes (black) in comparison to a positive control (red). Negative control mosquitoes start crossing the baseline threshold at cycle 38.74 when the baseline threshold is set at 230 RFU.
