# Supplementary material for: Vertical Transmission of Sindbis Virus in Culex Mosquitoes
Source: Viruses. 2022 Aug 30;14(9):1915. doi: 10.3390/v14091915 (PMC9504956; doi:10.3390/v14091915)
Supplement: Supplementary file 1 [file viruses-14-01915-s001.zip › viruses-1850058-supplementary.pdf]

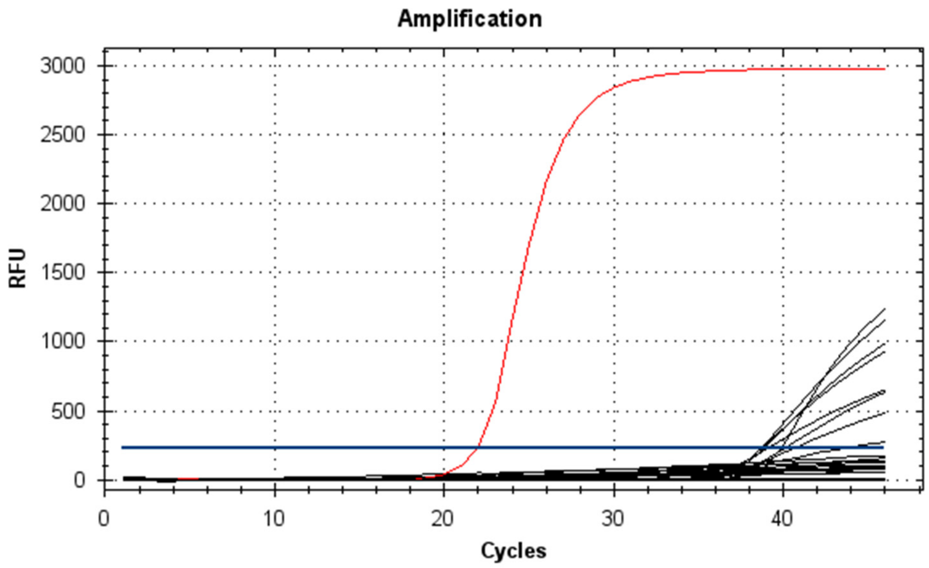

Figure S1. Amplification of uninfected negative control mosquitoes (black) in comparison to a positive control (red). Negative control mosquitoes start crossing the baseline threshold at cycle 38.74 when the baseline threshold is set at 230 RFU.
